# A multi-omics data analysis workflow packaged as a FAIR Digital Object

**DOI:** 10.1093/gigascience/giad115

**Published:** 2024-01-13

**Authors:** Anna Niehues, Casper de Visser, Fiona A Hagenbeek, Purva Kulkarni, René Pool, Naama Karu, Alida S D Kindt, Gurnoor Singh, Robert R J M Vermeiren, Dorret I Boomsma, Jenny van Dongen, Peter A C ’t Hoen, Alain J van Gool

**Affiliations:** Department of Medical BioSciences, Radboud University Medical Center, 6525 GA Nijmegen, The Netherlands; Translational Metabolic Laboratory, Department of Laboratory Medicine, Radboud University Medical Center, 6525 GA Nijmegen, the Netherlands; Department of Medical BioSciences, Radboud University Medical Center, 6525 GA Nijmegen, The Netherlands; Department of Biological Psychology, Vrije Universiteit Amsterdam, 1081 BT Amsterdam, The Netherlands; Amsterdam Public Health Research Institute, 1081 BT Amsterdam, The Netherlands; Department of Medical BioSciences, Radboud University Medical Center, 6525 GA Nijmegen, The Netherlands; Translational Metabolic Laboratory, Department of Laboratory Medicine, Radboud University Medical Center, 6525 GA Nijmegen, the Netherlands; Department of Human Genetics, Radboud University Medical Center, 6525 GA Nijmegen, The Netherlands; Department of Biological Psychology, Vrije Universiteit Amsterdam, 1081 BT Amsterdam, The Netherlands; Amsterdam Public Health Research Institute, 1081 BT Amsterdam, The Netherlands; Metabolomics and Analytics Centre, Leiden Academic Centre for Drug Research, Leiden University, 2333 AL Leiden, The Netherlands; Metabolomics and Analytics Centre, Leiden Academic Centre for Drug Research, Leiden University, 2333 AL Leiden, The Netherlands; Department of Medical BioSciences, Radboud University Medical Center, 6525 GA Nijmegen, The Netherlands; Department of Child and Adolescent Psychiatry, LUMC-Curium, Leiden University Medical Center, 2342 AK Oegstgeest, The Netherlands; Department of Biological Psychology, Vrije Universiteit Amsterdam, 1081 BT Amsterdam, The Netherlands; Amsterdam Public Health Research Institute, 1081 BT Amsterdam, The Netherlands; Amsterdam Reproduction & Development (AR&D) Research Institute, 1081 BT Amsterdam, The Netherlands; Department of Biological Psychology, Vrije Universiteit Amsterdam, 1081 BT Amsterdam, The Netherlands; Amsterdam Public Health Research Institute, 1081 BT Amsterdam, The Netherlands; Amsterdam Reproduction & Development (AR&D) Research Institute, 1081 BT Amsterdam, The Netherlands; Department of Medical BioSciences, Radboud University Medical Center, 6525 GA Nijmegen, The Netherlands; Translational Metabolic Laboratory, Department of Laboratory Medicine, Radboud University Medical Center, 6525 GA Nijmegen, the Netherlands

**Keywords:** multi-omics, workflow, metadata, FAIR, RO-Crate, FDO

## Abstract

**Background:**

Applying good data management and FAIR (Findable, Accessible, Interoperable, and Reusable) data principles in research projects can help disentangle knowledge discovery, study result reproducibility, and data reuse in future studies. Based on the concepts of the original FAIR principles for research data, FAIR principles for research software were recently proposed. FAIR Digital Objects enable discovery and reuse of Research Objects, including computational workflows for both humans and machines. Practical examples can help promote the adoption of FAIR practices for computational workflows in the research community. We developed a multi-omics data analysis workflow implementing FAIR practices to share it as a FAIR Digital Object.

**Findings:**

We conducted a case study investigating shared patterns between multi-omics data and childhood externalizing behavior. The analysis workflow was implemented as a modular pipeline in the workflow manager Nextflow, including containers with software dependencies. We adhered to software development practices like version control, documentation, and licensing. Finally, the workflow was described with rich semantic metadata, packaged as a Research Object Crate, and shared via WorkflowHub.

**Conclusions:**

Along with the packaged multi-omics data analysis workflow, we share our experiences adopting various FAIR practices and creating a FAIR Digital Object. We hope our experiences can help other researchers who develop omics data analysis workflows to turn FAIR principles into practice.

Key Points

- The FAIR4RS principles provide guidelines to enhance the discovery and reuse of research software.
- FAIR Digital Objects support Findability, Accessibility, Interoperability, and Reusability by both humans and machines.
- We here demonstrate an implementation of a multi-omics data analysis workflow and share it as a FAIR Digital Object.

## Background

The FAIR principles for research data [1] were proposed to guide researchers to create research data that is Findable, Accessible, Interoperable, and Reusable (FAIR). Since these guidelines aim to enable researchers handling and navigating through the rapidly increasing amounts of data, special emphasis was put on concepts to make data not only usable by humans but also machine-actionable. In the past years, various standards [2, 3] and implementations [4–7] of the FAIR principles have been introduced, and it has been demonstrated that FAIR data are beneficial to research and patients [8–10]. Reuse of research data and reproducibility of research results [11] are facilitated by good data provenance, and this requires not only the data but also the data processing and analysis workflows to be FAIR. Consequently, guidelines and practices for FAIR research software have been proposed [12–14] (see Table 1), and the special case of computational workflows has been discussed [15, 16]. These guidelines aim to increase reproducibility not only at the experimental level but also at the data analysis level. It has been shown that the availability of data and code alone is not sufficient. They both need to be provided in an open and interoperable format and described by metadata [17].

**Table 1:** Overview of recommended FAIR practices for research data and software

FAIR guiding principles [1]	Open-source software recommendations [12]	Recommendations for FAIR software [13]	FAIR principles for research software [14, 18]
**Findable**			F. Software, and its associated metadata, is easy for both humans and machines to find.
F1. (Meta) data are assigned globally unique and persistent identifiers.			F1. Software is assigned a globally unique and persistent identifier.
			F1.1. Components of the software representing levels of granularity are assigned distinct identifiers.
			F1.2. Different versions of the software are assigned distinct identifiers.
F2. Data are described with rich metadata.			F2. Software is described with rich metadata.
F3. Metadata clearly and explicitly include the identifier of the data they describe.			F3. Metadata clearly and explicitly include the identifier of the software they describe.
F4. (Meta)data are registered or indexed in a searchable resource.	R2. Make software easy to discover by providing software metadata via a popular community registry.	#3 Register your code in a community registry.	F4. Metadata are FAIR, searchable, and indexable.
**Accessible**	R1. Make source code publicly accessible from day 1.	#1 Use a publicly accessible repository with version control.	A. Software, and its metadata, is retrievable via standardized protocols.
A1. (Meta)data are retrievable by their identifier using a standardized communication protocol.			A1. Software is retrievable by its identifier using a standardized communications protocol.
A1.1. The protocol is open, free, and universally implementable.			A1.1. The protocol is open, free, and universally implementable.
A1.2. The protocol allows for an authentication and authorization procedure where necessary.			A1.2. The protocol allows for an authentication and authorization procedure, where necessary.
A2. Metadata should be accessible even when the data are no longer available.			A2. Metadata are accessible, even when the software is no longer available.
**Interoperable**			I. Software interoperates with other software by exchanging data and/or metadata, and/or through interaction via application programming interfaces (APIs), described through standards.
I1. (Meta)data use a formal, accessible, shared, and broadly applicable language for knowledge representation.			I1. Software reads, writes, and exchanges data in a way that meets domain-relevant community standards.
I2. (Meta)data use vocabularies that follow the FAIR principles.			
I3. (Meta)data include qualified references to other (meta)data.			I2. Software includes qualified references to other objects.
**Reusable**	R4. Define clear and transparent contribution, governance, and communication processes.	#4 Enable citation of the software; #5 Use a software quality checklist.	R. Software is both usable (can be executed) and reusable (can be understood, modified, built upon, or incorporated into other software).
R1. (Meta)data are richly described with a plurality of accurate and relevant attributes.			R1. Software is described with a plurality of accurate and relevant attributes.
R1.1. (Meta)data are released with a clear and accessible data usage license.	R3. Adopt a license and comply with the license of third-party dependencies.	#2 Add a license.	R1.1. Software is given a clear and accessible license.
R1.2. (Meta)data are associated with detailed provenance.			R1.2. Software is associated with detailed provenance.
			R2. Software includes qualified references to other software.
R1.3. (Meta)data meet domain-relevant community standards.			R3. Software meets domain-relevant community standards.

Several practices recommended for research software development originate from general software engineering practices [12, 15, 19], which include version control, documentation, and licensing. Version control of source code facilitates collaborative development and monitoring changes [13]. Additionally, making the code publicly available on dedicated software repositories that support version control such as Git-based [20] GitHub [21], GitLab [22], or BitBucket [23] contributes to findability [24], accessibility [12], and reusability [13]. The documentation of research software includes multiple levels. First, a comprehensive end-user documentation and usage examples enable reusability by other researchers [17, 24–26]. It should also include the documentation of workflow parameters [16, 17]. Second, source code documentation enables other developers to understand and build upon the software [17]. Documentation of code changes via a version control system helps document the development process [19, 25], and documentation of dependencies is prerequisite for software interoperability [24] and reusability [18]. Adding a clear and machine-readable [16] license is essential to allow for software reuse. It is recommended to choose a widely used and preferably open-source license that is compatible with licenses of the dependencies [12–14, 18, 19, 24, 25]. Examples of open-source licenses with few restrictions are the Apache License 2.0 [27] and the MIT License [28].

There are differences between research software that implements a specific method as a standalone tool or a software library and complex analysis workflows [16]. Computational analysis workflows can comprise numerous steps that are integrated into pipelines [16] and are often developed in a specific project [19, 29]. With a multitude of analysis steps being combined into complex workflows, the documentation of the individual analyses and their dependencies can become challenging. To facilitate the automation of analysis tasks and their documentation, workflows can be described using workflow management systems such as Nextflow [30] or Snakemake [31]. Workflow managers that support the creation of reusable modules can help reduce complexity and promote the reuse of workflows or workflow modules [15, 16, 32]. Additionally, notebooks can apply the concept of literate programming and are a useful tool to add human-readable documentation next to code blocks [19]. Interoperability and reusability of workflows can be achieved using portable software containers such as Apptainer/Singularity [33] or Docker [34] that capture the runtime environment of a workflow or a workflow module [15, 16, 26, 35].

Computational workflows can be regarded as digital objects. The concept of FAIR Digital Objects (FDOs) was introduced to make digital objects fully FAIR [36]. FDOs comprise, among others, the digital object, a persistent identifier (PID), and metadata (title, authors, licenses, etc.) describing the object. The RO-Crate approach was specified to package digital research artifacts or Research Objects (ROs) such as computational workflows [37]. The RO-Crate contains a PID that links to an RO, which is described by a structured JSON-LD RO-Crate metadata file. It contains all contextual and noncontextual related data to rerun the workflow. In case the actual data cannot be publicly shared due to privacy reasons, synthetic data can complement analysis workflows to demonstrate the computational procedure [16, 38]. To make an RO-Crate findable, it needs to be registered at a registry such as WorkflowHub [39, 40]. The WorkflowHub RO-Crate represents an approach to implementing the FDO concept [41,42].

We here demonstrate the development of a FAIR Digital Object comprising a computational workflow that analyzes and integrates multi-omics and phenotype data and is associated with rich human and machine-readable metadata.

## Findings

### Workflow implementation

**Figure 1:**
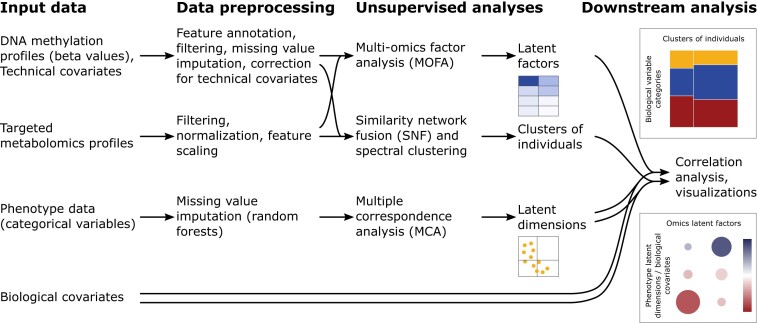
Overview of analysis steps.

To develop a reusable workflow, our input data and intermediate files were largely based on open and widely used formats or community standards. For the metabolomics data and metadata, we adopted practices of the MetaboLights database [43] of the European Bioinformatics Institute (EBI) of the European Molecular Biology Laboratory (EMBL). Metabolite levels and annotations are reported in metabolite annotation/assignment files (MAFs). The experimental metadata for omics measurements are reported using the Investigation/Study/Assay (ISA) metadata framework [44]. We employed Jupyter [45] and the Python ISA API [46] to create ISA-Tab and ISA-JSON files [47]. For machine-readable descriptions of the experiments, ontology terms were used. Ontologies are standardized taxonomies of entities of a specific subject (domain), including definitions of relationships between these entities. Ontology terms refer to these entities [48]. Based on recommended standards from FAIRgenomes [3] and Metabolights [43], we preferably employed the following ontologies: National Cancer Institute Thesaurus (NCIT) [49], Experimental Factor Ontology (EFO) [50], Ontology for Biomedical Investigations (OBI) [51], Metabolomics Standards Initiative Ontology (MSIO) [52], Chemical Methods Ontology (CHMO) [53], and Chemical Entities of Biological Interest (ChEBI) [54]. The DNA methylation levels and associated metadata, behavioral data, and additional information about phenotypes or technical and biological covariates are stored as comma-separated value (CSV) files. This allows our computational workflow to be easily reusable and adaptable for other datasets. The workflow documentation  [55] describes all input files used in the workflow and provides human-readable descriptions of every step of the workflow processing and integrating individual input data types. Each of these analysis steps (see Figure 1) is implemented in Python or R and added as a module to the workflow. We employ Jupyter and R notebooks for implementing downstream analyses and visualization of results. We chose Nextflow as our workflow management system, since it allows flexible development, can be run on different platforms, supports containers, is well documented, and is already widely adopted by the bioinformatics community [32]. Each module of the workflow is provided with their own Docker container to ensure portability and eliminate the need for local software installations.

**Figure 2:**
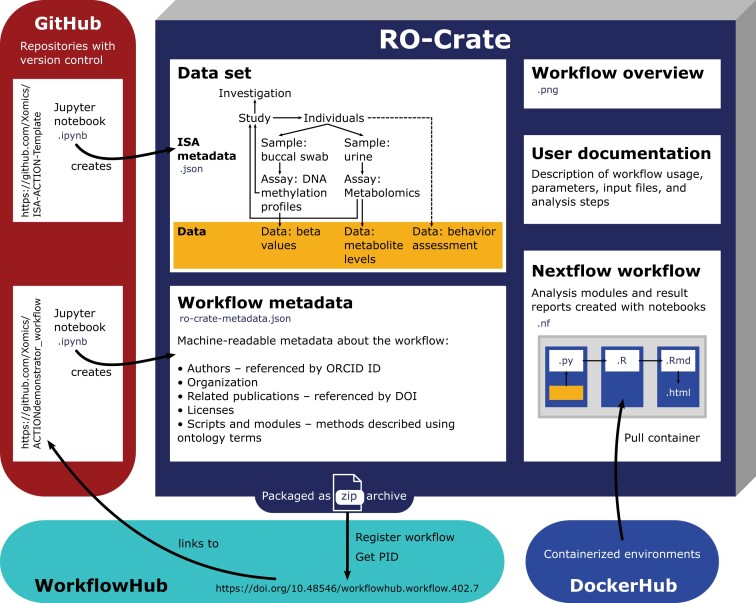
Schematic overview of packaged workflow.

Finally, the Nextflow workflow is packaged as an RO-Crate. In addition to the workflow and a synthetic dataset, it contains a structured metadata file with machine-readable descriptions of input files and analysis steps (ro-crate-metadata.json). We preferably used EDAM–Ontology of bioscientific data analysis and data management [56] as it is recommended for workflow RO-Crates [37]. For terms that were not available in EDAM, alternative ontologies such as NCIT [49], OBI [51], or the Semanticscience Integrated Ontology (SIO) [57] were used. We employed the Python package ro-crate-py [58] to create the RO-Crate metadata file. The RO-Crate further contains an image with an overview of the analysis steps. For findability, the packaged workflow (see Fig. 2) is registered on WorkflowHub [39] and provided with a Digital Object Identifier (DOI) (https://doi.org/10.48546/workflowhub.workflow.402.8).

### Case study

Our workflow was developed to analyze and integrate DNA methylation and urine metabolomics profiles with behavioral data originating from the ACTION Biomarker Study (ACTION, Aggression in Children: Unraveling Gene-Environment Interplay to Inform Treatment and Intervention strategies) [59–61] (see “Case Study” in the Methods section). Within ACTION, urine and buccal cell samples were collected in a twin cohort from the Netherlands Twin Register (NTR) and in a cohort of children referred to an academic center for child and youth psychiatry in the Netherlands (LUMC-Curium). These children were also characterized for behavioral problems, and here we look at externalizing problems. We purposely selected a case of complex human behavioral phenotype that is typically not caused by a single well-defined molecular defect but originates from changes in multiple factors and as such would benefit from a multi-omics analysis. Since we consider these data to be potentially personally identifiable information, we share a synthetic dataset to demonstrate the workflow. The goal of the analysis is the identification of substructures in the multi-omics data and to determine if they correlate with behavioral data (see “Unsupervised Data Analysis”). A team comprising members of the Netherlands X-omics Initiative [62] in collaboration with the NTR [63] developed the computational workflow. To uncover possible relationships between the multi-omics data and the behavioral data, we applied different unsupervised data-driven methods followed by downstream analyses, including determining the effect of possible confounding factors of sex and age. An overview of the main analysis steps is shown in Fig. 1. An overview of data dimensions and types during different steps of the workflow is provided in Additional File 10.

To identify underlying patterns in childhood externalizing behavior, we applied multiple correspondence analysis (MCA)  [64, 65] to the parent-rated responses on the externalizing behavior items of the Child Behavior Checklist (CBCL) of the Achenbach System of Empirically Based Assessment (ASEBA) [66] in both cohorts. In NTR participants, the first 3 MCA dimensions jointly explain 30% of the variation in 26 externalizing behavior items of the ASEBA CBCL (see Additional File 1). Additional dimensions each explain <5% of the variation. The presence rather than the absence of externalizing behaviors characterized all of the first 3 dimensions, which reflects the answer options to items (a problem behavior is not present, a little, or a lot). Variables that contributed most to the first dimension, which explained 16% of the variation, represent temperamental behavior (frequent temper tantrums, stubbornness, screaming, and arguing). Variables contributing to the second dimension, which explained 9% of the variation, represent hostile aggressive behaviors (frequent vandalism, bullying, and cruelty). In LUMC-Curium participants, the first 2 MCA dimensions suffice to explain 30% of the variation in 18 items of the ASEBA CBCL (see Additional File 2). Similar to NTR, these first dimensions in LUMC-Curium are characterized by the presence of aggressive behaviors.

We applied multi-omics factor analysis (MOFA) [67] in both cohorts to obtain 10 factors to describe the buccal DNA methylation (Illumina EPIC array) and urine metabolomics data. For this analysis, we selected the top 10% most variable probes from DNA methylation data. Cumulatively, the 10 factors explained 22.5% and 74.9% of variation in the DNA methylation data and 0.001% and 1.89% in the urine metabolomics data in NTR (see Additional File 3) and LUMC-Curium (see Additional File 4), respectively. We observed no evidence that any of the factors captured sources of variation in both the DNA methylation and urine metabolomics data in NTR and LUMC-Curium. In particular, factors 1 and 2 in NTR and factor 1 in LUMC-Curium were specific to the DNA methylation data. To help elucidate the etiology of the 10 MOFA factors, we selected for each factor the top 100 CpGs with the largest weights and performed enrichment analyses within the Epigenome-Wide Association Study (EWAS) atlas [68]. Multiple factors in both cohorts (see Additional File 5 for ACTION-NTR and Additional File 6 for LUMC-Curium cohort) showed enrichment of CpGs associated with glucocorticoid exposure (i.e., administration of corticosteroid medication [69]), CpGs associated with aging, and CpGs associated with immune-related traits, such as psoriasis. Apart from these robustly enriched traits, additional significant enrichments were found but were often based on ≤5 overlapping CpGs between the factor results and the original studies. A limitation of the enrichment analysis is that the most previous EWAS studies included in this analysis were conducted on blood samples from adult populations with the Illumina 450K BeadChip. In the factor weights for metabolites, we observe that for both NTR (Additional File 3) and LUMC-Curium (Additional File 4), many of the factors are characterized by only 1 or few metabolites. We note that in both cohorts, the factors explained only a small amount of variation in the metabolomics data. To investigate whether the omics factors were associated with behavioral dimensions (MCA), we ran generalized estimation equation (GEE) models adjusting for relatedness in NTR and correlation analyses in Curium (see Additional File 3 for ACTION-NTR and Additional File 4 for LUMC-Curium cohort). None of the omics factors were significantly associated with the behavioral dimensions in NTR or LUMC-Curium, nor did we observe significant associations of sex- and age-specific T-scores for aggressive behavior with the omics factors. In previous multi-omics analyses of high versus low levels of childhood aggression [70] and attention-deficit/hyperactivity disorder (ADHD) [71], we applied supervised analyses in these cohorts while applying unsupervised analyses here. In these previous supervised analyses, where we also included an additional omics layer—polygenic scores—we found that although multi-omics models had low predictive value, they revealed some connections of omics traits with externalizing problems, which suggested biological plausibility.

We also constructed integrated similarity networks with Similarity Network Fusion (SNF)  [72] to identify subgroups of individuals based on omics data. In both NTR and LUMC-Curium, we defined integrated similarity networks based on 2 and 4 clusters. The 2 clusters in NTR were characterized by differences in age, whereas the 2 clusters in LUMC-Curium were characterized by differences in the proportion of boys and girls. To investigate whether the omics clusters were associated with externalizing behavior, we compared the behavioral dimension scores from MCA between children in the different clusters. In both NTR and LUMC-Curium, we observed no significant differences in the behavioral dimensions across the 2 omics clusters after correction for multiple testing (see Additional File 7 for NTR and Additional File 8 for LUMC-Curium cohort). Similarly, no differences in behavioral dimensions were observed between the 4 omics clusters in NTR, but in LUMC-Curium, behavioral dimension 6 differed significantly between the 4 omics clusters. In LUMC-Curium, dimension 6 explained 3.9% of the variance in childhood externalizing behavior, and the strongest contributors to this dimension comprised higher frequencies of parent-rated tendencies to be suspicious and loud. Such forms of direct aggressive behavior, particularly physical aggressive behavior, are common in early childhood in both boys and girls, and while overall levels of aggression decline with age and are roughly similar for boys and girls [73], boys are more likely to engage in direct and physical forms of aggression by age 11 [74]. Thus, this finding aligns with the observation that the 2 omics clusters differ in the proportion of males and females and in the age composition.

Our data-driven approach to identifying possible relationships between multi-omics and behavioral data did not reveal significant findings that could not also be explained by potential confounding factors of sex or age. Since we here focused on latent dimensions representing the largest variations between individuals (after correcting for known confounders), it is possible that relationships between omics and aggressive behavior can be found in lower dimensions that reflect only a small amount of variation in the cohorts. However, including more (latent) variables in the correlation analysis will also increase the chance of false-positive findings.

## Discussion

In this collaborative research project, partners from the Netherlands X-omics Initiative codeveloped a workflow to analyze a complex multimodal dataset. Developing workflows with partners across multiple institutions can pose a challenge, and we experienced that a secure shared computing environment was key to the success of this project. Additionally, practices aiming to increase FAIRness of the shared workflow such as version control with Git and a modular workflow structure allowed for transparent and target-oriented workflow development. Therefore, while the use of technologies like Git or workflow management systems might require initial training of researchers, we believe this to be worthwhile not only for future reuse but also during workflow development.

To make the workflow findable, we registered it in WorkflowHub [39], which is part of the European Open Science Cloud (EOSC) [75]. Since this was the first workflow we registered in WorkflowHub, we profited from its documentation and active community. The registry allowed us to assign a globally unique and persistent identifier to the workflow [76] and its versions. Metadata could be added using the open RO-Crate standard and are searchable in the registry. The workflow page [76] links to the publicly accessible and version-controlled source code on GitHub [77].

Several FAIR practices for workflows include existing best practices of software development, for example, version control and good documentation. Adoption of these practices, along with the use of workflow managers and software containers, aims to contribute to better interoperability, reusability, and reproducibility of analysis workflows and research results. While we experienced the adoption of these technologies to be straightforward, fully FAIR, and especially interoperable, data or software requires also machine-understandable semantic metadata. Specifications like the ISA metadata framework and RO-Crate allow ontology-based annotations of omics experiments and analysis workflows, respectively. Our choice of ontologies was mainly guided by the documented submission requirements or recommendations provided by services such as the MetaboLights archive or WorkflowHub. However, when recommended ontologies do not comprise suitable terms, choosing appropriate ones from ontologies can be challenging. For example, no exact match to the generic term *sample collection* that is part of the ISA schema can be found in any ontology available in EBI’s Ontology Lookup Serivce (OLS) [78]. To describe workflow steps in RO-Crate with *unsupervised learning*, we had to employ the eNanoMapper Ontology [79] as no matching term was available in the recommended EDAM ontology. Consequently, we recognize the importance of teams dedicated to ontology curation, active user communities, and training of researchers in using semantic technologies. This is especially important for multi-omics research that spans multiple research domains.

While machine actionability supported by standardized metadata is relevant for interoperability, the workflow also needs to be usable and reusable by humans. We added software containers that are referenced by the workflow metadata. They enable portability and thereby reusability. A user documentation was added to help understand the workflow steps and facilitate reuse. Enabling richer workflow annotation with RO-Crate in combination with additional tooling that enable automated generation of user documentation could potentially reduce the efforts of manual workflow documentation in the future.

For reproducibility of research results, it is essential that data are shared along with the workflow. However, privacy regulations prohibit sharing of potentially personally identifiable data such as omics measurements or clinical information. To demonstrate the functionality of the workflow, we shared a synthetic dataset that emulates the structure of the case study dataset. Current developments in the areas of federated data storage and analysis such as Federated European Genome-Phenome Archive (EGA) [80] and the Personal Health Train [81] have the potential to allow fully FAIR and reproducible data analysis workflows while maintaining privacy regulation compliance.

Implementing these FAIR practices required us to use various tools, some of which we used for the first time. While this required some time and openness to getting familiarized with these tools, we experienced that the tools were generally well documented and could quickly be adopted. Open online resources such the ELIXIR’s [82] community-driven FAIR Cookbook [83, 84] provide guides and examples that can help researchers implement FAIR practices. Existing Python libraries such as the ISA API[46] and ro-crate-py [58] were very useful when implementing metadata standards as they can help ensure compliance with the standards as well as automating creation of metadata files. However, it would have been useful if more use cases implementing FAIR practices for scientific computational workflows were available as examples or tutorials. We experienced that implementing FAIR practices from the start helped us create a transparent multi-omics analysis workflow. Additionally, we are convinced that FAIR workflows are key to not only reproducible but also efficient research as workflows or subworkflows can be reused in new contexts, thereby saving time. Therefore, we hope our experiences help other researchers who develop multi-omics data analysis workflows choosing and implementing practices that makes their research more FAIR.

## Data and Methods

### Case study

Our case study comprises data from 2 cohorts that took part in the ACTION Biomarker Study [59–61]. The ACTION Biomarker Study collected buccal DNA samples for large-scale genome-wide and epigenome-wide association studies [85, 86] and first-morning urine samples to investigate the association of urine biomarkers and metabolites with childhood aggression [61]. These urine and buccal cell samples were collected in a twin cohort from the NTR [87], where twin pairs were selected on their longitudinal concordance or discordance for childhood aggression, and in a cohort of children referred to an academic center for child and youth psychiatry in the Netherlands (LUMC-Curium). The DNA methylation, genotype, metabolomics, and behavioral data from these cohorts were previously used for multi-omics analyses of aggressive behavior [70] and ADHD [71]. Detailed information on the study populations and study protocol is available at protocols.io [88].

#### Data

Genome-wide DNA methylation data in buccal DNA samples were measured on the Infinium MethylationEPIC BeadChip kit (Illumina [89]) by the Human Genotyping Facility (HuGe-F) of ErasmusMC (the Netherlands [90]). The ZymoResearch EZ DNA Methylation kit (Zymo Research Corp) was used for bisulfite treatment of 500 ng enomic DNA obtained from buccal swabs. The Infinium HD Methylation Assay was performed according to the manufacturer’s specification. Good Biomarker Sciences Leiden measured the specific gravity (by refractomertry), levels of creatinine (by colorimertry), blood traces, markers of leukocytes, proteins, glucose, and nitrites (the latter 5 by dipstick) of each urine sample. The Metabolomics Facility of the University of Leiden quantified urine metabolites using 3 platforms: a liquid chromatography–mass spectrometry (LC-MS) platform targeting amines (66 biomarkers), an LC-MS platform targeting steroid hormones (13 biomarkers), and a gas chromatography–mass spectrometry (GC-MS) platform targeting organic acids (21 biomarkers). Behavioral data comprise the 115 items of the Dutch version of the ASEBA CBCL for school-aged children (6–18 years) [66]. For participants of the NTR cohort, we used the mother-rated CBCL as completed at the time of biological sample collection, and for participants of the LUMC-Curium cohort, we used the parent-rated (90% mother ratings) CBCL as completed in a 6-month window surrounding the biological sample collection. Again, details on the data generation are available in [88].

#### Synthetic data and metadata

The purpose of the synthetic dataset that is part of the RO-Crate is to demonstrate how the workflow can be run. It resembles the structure of the files of the cohort data. The values were randomly sampled from the observed values in the NTR cohort without preserving any correlations. While creation of ISA metadata is not part of this workflow, we share the Jupyter notebook employing the Python ISA API [46] that was used to create the metadata for the synthetic dataset [47].

### Data processing

To ensure the urine sample metabolic integrity and to minimize bias contributed by health conditions, we excluded samples from the metabolomics data from (1) subjects who have started menstruating, (2) subjects in whom the time between urine sample collection and storing in the freezer was >2 hours, (3) subjects in whom severe violations to the sampling protocol occurred (e.g., not putting a lid on the container), (4) subjects in whom the leukocyte count was above trace, (5) subjects in whom the nitrites level was “positive high,” (6) subjects in whom the protein level was >0.3, (7) subjects with glucose levels above trace, (8) subjects with blood levels above trace, (9) subjects having the flu, (10) subjects reporting inflammation, (11) subjects reporting vomiting, (12) subjects reporting abdominal pain, and (13) subjects reporting general health problems. Note that the above criteria 4–8 are based on the dipstick marker estimation performed separately from the metabolomics measurements on the same samples [88], while the other criteria are based on questionnaire data at the time of sampling.

The metabolomics features were filtered based on missing values. Missing values were reported for cases where the metabolite concentration is below the limit of quantification. Samples and metabolites with 15% or more of missing values were discarded. Sample-wise normalization to correct for urine concentration was conducted by adjusting metabolite intensities to the sample creatinine levels [88]. This was followed by metabolite-wise Pareto scaling [91] to statistically account for large differences in reported values.

Quality control (QC) and normalization of the DNA methylation array data have been previously described [85] and were carried out with a pipeline developed by the Biobank-based Integrative Omics Study (BIOS) consortium [92]. From the 787,711 autosomal methylation probes that survived QC, the top 10% most variable probes were included in the analyses. Cellular proportions of buccal samples were predicted with Hierarchical Epigenetic Dissection of Intra-Sample-Heterogeneity (HepiDISH) with the reduced partial correlation (RPC) method, as described  Zheng et al. [93] and implemented in the R/Bioconductor package EpiDISH. Median imputation was carried out on the epigenetics data. Residual methylation levels were obtained by regressing the effects of percentages of epithelial and natural killer cells, EPIC array row, and bisulfite sample plate from the methylation beta-values.

Missing values in the externalizing behavior items were imputed with the nonparametric random forests method from the R library missForest (1.4) [94].

### Unsupervised data analysis

Each cohort was analyzed separately. We applied MOFA using the R/Bioconductor library MOFA2 (1.3.4) [67, 95] to obtain factors for the buccal DNA methylation and urine metabolomics data and applied MCA [65] using the R library FactoMineR (2.4) [64] to obtain factors for the behavioral data.

To identify subgroups of individuals based on their buccal DNA methylation and urine metabolomics data, we constructed integrated similarity networks with SNF [72]. The optimal numbers of clusters were determined using a built-in function of the Python library SNFpy [96] that uses the eigengap method [97] to find the optimal number of clusters. SNF first constructs sample similarity networks for each available data type and then fuses these into a single network comprising both the shared and unique information from each data type. The final fused network thus captures how each data type contributes to the similarity among the samples. We tested whether the behavioral dimension scores from MCA differ between children in the different SNF clusters, using Mann–Whitney *U* tests (2 clusters) or Kruskal–Wallis tests (four clusters) in the Curium cohort, and with GEE models (with cluster as predictor and behavioral dimension score as outcome) in NTR.

We determined correlations among the obtained factors capturing the omics and behavioral data, respectively, using Spearman’s rank correlation and additionally in the NTR cohort using GEE models. All GEE models were fitted with the R package GEE, with the following specifications: Gaussian link function (for continuous data), 100 iterations, and the “exchangeable” option to account for the correlations in twin pairs. Statistical tests were adjusted for multiple testing using the false discovery rate [98].

## Availability of source code and requirements

- Project name: X-omics ACTION demonstrator multi-omics analysis workflow
- Project homepage: [77]
- Operating system(s):  Platform independent
- Programming language:  Python, R
- Other requirements: Nextflow (22.04.0), Docker (19.03.1), Singularity (3.8.0)
- License:  MIT
- SciCrunch: RRID:SCR_024719

## Supplementary Material

giad115_GIGA-D-23-00162_Original_Submission

giad115_GIGA-D-23-00162_Revision_1

giad115_GIGA-D-23-00162_Revision_2

giad115_Response_to_Reviewer_Comments_Original_Submission

giad115_Response_to_Reviewer_Comments_Revision_1

giad115_Reviewer_1_Report_Original_Submission Carole Goble -- 7/13/2023 Reviewed

giad115_Reviewer_2_Report_Original_Submission Dominique Batista -- 7/26/2023 Reviewed

giad115_Reviewer_3_Report_Original_Submission Megan Hagenauer, Ph.D. -- 8/31/2023 Reviewed

giad115_Supplemental_Files

## Data Availability

Details on data availability can be found in Additional File 10. The data of the Netherlands Twin Register (NTR) ACTION Biomarker Study may be accessed, upon approval of the data access committee, through the NTR [99]. A synthetic dataset representing the structure of the ACTION Biomarker Study dataset is available as part of the workflow RO-Crate available at WorkflowHub [76]. An archival copy of the workflow is also available via the *GigaScience* database, GigaDB [100].

## Additional Files

- File name: Additional File 1
- File format: .html
- Title: Multiple Correspondence Analysis of CBCL Behavioral Data
- Description: Overview of externalizing behavior items of the Child Behavior Checklist (CBCL) of the Achenbach System of Empirically Based Assessment (ASEBA) in the ACTION-NTR cohort before and after imputation of missing values using random forests and results and visualizations of multiple correspondence analysis (MCA).

- File name: Additional File 2
- File format: .html
- Title: Multiple Correspondence Analysis of CBCL Behavioral Data
- Description: Overview of externalizing behavior items of the Child Behavior Checklist (CBCL) of the Achenbach System of Empirically Based Assessment (ASEBA) in the LUMC-Curium cohort before and after imputation of missing values using random forests and results and visualizations of multiple correspondence analysis (MCA).

- File name: Additional File 3
- File format: .html
- Title: MOFA Downstream Analysis Report
- Description: Visualizations of multi-omics factor analysis (MOFA) of buccal DNA methylation (Illumina EPIC array) and urine metabolomics data of the ACTION-NTR cohort. Also, associations between the MOFA factors and phenotypic data are tested with GEE models.

- File name: Additional File 4
- File format: .html
- Title: MOFA Downstream Analysis Report
- Description: Visualizations of multi-omics factor analysis (MOFA) of buccal DNA methylation (Illumina EPIC array) and urine metabolomics data of the LUMC-Curium cohort.

- File name: Additional File 5
- File format: .xlsx
- Title: EWAS Atlas Enrichment Analysis
- Description: Enriched traits for CpGs with top 100 largest weights of ACTION-NTR MOFA factors 1 to 10.

- File name: Additional File 6
- File format: .xlsx
- Title: EWAS Atlas Enrichment Analysis
- Description: Enriched traits for CpGs with top 100 largest weights of LUMC-Curium MOFA factors 1 to 10.

- File name: Additional File 7
- File format: .html
- Title: Similarity Network Fusion Downstream Analysis
- Description: Visualizations of similarity network fusions (SNFs) and subsequent spectral clustering of buccal DNA methylation (Illumina EPIC array) and urine metabolomics data of the ACTION-NTR cohort.

- File name: Additional File 8
- File format: .html
- Title: Similarity Network Fusion Downstream Analysis
- Description: Visualizations of similarity network fusions (SNFs) and subsequent spectral clustering of buccal DNA methylation (Illumina EPIC array) and urine metabolomics data of the LUMC-Curium cohort.

- File name: Additional File 9
- File format: .html
- Title: Similarity Network Fusion Downstream Analysis With GEE models
- Description: Associations between the similarity network fusion (SNF) clusters and phenotypic data are tested with GEE models in the ACTION-NTR cohort.

- File name: Additional File 10
- File format: .pdf
- Title: Overview of Data Dimensions for ACTION-NTR and LUMC-CURIUM Cohort
- Description: Table with dimensions (variables by observations) of input and intermediate data types.

## Abbreviations

ACTION: Aggression in Children: Unraveling Gene-Environment Interplay to Inform Treatment and Intervention Strategies; ASEBA: Achenbach System of Empirically Based Assessment; EBI: European Bioinformatics Institute; EGA: European Genome-Phenome Archive; EMBL: European Molecular Biology Laboratory; EWAS: epigenome-wide association study; FAIR: Findable, Accessible, Interoperable, Reusable; GC-MS: gas chromatography–mass spectrometry; ISA: Investigation, Study, Assay; LC-MS: liquid chromatography–mass spectrometry; MOFA: multi-omics factor analysis; NTR: Netherlands Twin Register; RO-Crate: Research Object Crate; SNF: Similarity Network Fusion.
