# Supplementary material for: A multi-omics data analysis workflow packaged as a FAIR Digital Object
Source: Gigascience. 2024 Jan 13;13:giad115. doi: 10.1093/gigascience/giad115 (PMC10787363; doi:10.1093/gigascience/giad115)
Supplement: giad115_Supplemental_Files [file giad115_supplemental_files.zip › Additional File 10.pdf]

Supplementary Table 1: Overview of data dimensions (variables × observations) for ACTION-NTR and LUMC-CURIUM (values are given in parentheses) cohort.

| | **Multi-omics data (numeric)** | | | | | **Phenotype (behavioral) data (categorical)** | **Biological covariates (categorical)** |
|---|---|---|---|---|---|---|---|
| | Targeted metabolomics profiles | | | DNA methylation profiles (beta values) | | | |
| | Amines | Organic acids | Steroid hormones | | | | |
| **Input data dimensions** | 64 × 1110<br>(64 × 169) | 20 × 1110<br>(20 × 169) | 11 × 1110<br>(11 × 169) | 857793 × 1237<br>(857793 × 186) | Technical covariates<br>• Sample plate (categorical)<br>• Arrow row number (numeric)<br>• Epithelial cell counts (numeric)<br>• Natural killer (NK) cell counts (numeric) | 26 × 1504<br>(18 × 189) | n/a |
| **Data dimensions after preprocessing, i.e., input for unsupervised analyses** | 61 × 1109<br>(60 × 169) | 20 × 1107<br>(20 × 169) | 11 × 1107<br>(11 × 169) | 69300 × 1237<br>(78781 × 186) | | 26 × 1349<br>(18 × 185) | n/a |
| **Dimensions used in downstream analyses** | MOFA<br>• 10 latent dimensions × 931<br>(10 × 167)<br><br>SNF fused networks (samples x samples)<br>• 931 × 931<br>(167 × 167)<br><br>SNF clusters<br>• 2 clusters x 931<br>(2 × 167)<br>• 4 clusters × 931<br>(4 × 167) | | | | | MCA<br>• 10 latent dimensions × 1349<br>(10 × 185) | • Age<br>• Sex<br>• Sick<br>• Menstruation<br>• Vitamin use |
