# Supplementary material for: A multi-omics data analysis workflow packaged as a FAIR Digital Object
Source: Gigascience. 2024 Jan 13;13:giad115. doi: 10.1093/gigascience/giad115 (PMC10787363; doi:10.1093/gigascience/giad115)
Supplement: giad115_Supplemental_Files [file giad115_supplemental_files.zip › Additional File 9.html]

SNF downstream analysis with GEE


# SNF downstream analysis with GEE

#### Casper de Visser1

### Load SNF dataframe

```
print(params$input_file_snf_pheno)
```

```
## [1] "snf_analysis_out.csv"
```

```
df_snf_phenotypes <- read.csv(params$input_file_snf_pheno, row.names=1)
df_snf_phenotypes <- df_snf_phenotypes[order(df_snf_phenotypes$Familynumber),]
```

### GEE model to correct for twin effect

```
library(gee)


#' Function for GEE model
#'
#' @param data Dataframe with pheno metadata
#' @param pheno_feature Feature to select as outcome
#' @param family Gaussian/Binomial
#' @return GEE model
geemodel <- function (data, pheno_feature, snf_label, family) {

f <- as.formula(
    paste(pheno_feature,
        paste(snf_label),
        sep = " ~ "))

invisible(gee(f, data=data, id=Familynumber, family=family, corstr="exchangeable", maxiter=100, na.action=na.omit, silent=T))
}


#' Function to run the GEE model with mofa factors as predictor for phenotypic feature
#'
#' @param data Dataframe with pheno metadata
#' @param pheno_feature Feature to select as outcome
#' @param family Gaussian/Binomial
#' @return end table with estimates and p-values
run_geemodel <- function(data, pheno_feature, snf_label, family=gaussian) {


# Make dataframes for storing statistics
estimates <- matrix(NA, 10, 2)
robustSE <- matrix(NA, 10, 2)
robustZ <- matrix(NA, 10, 2)
pval <- matrix(NA, 10, 2)


coeff <- summary(invisible(geemodel(data, pheno_feature, snf_label, family)))$coefficients
coeff <- data.frame(coeff)
coeff$pval <- 2*pnorm(-abs(coeff[,5]))
coeff$padjusted <- p.adjust(coeff$pval)

return(coeff)

}


### Set categorial variables to factors
```

```
df_snf_phenotypes$SNF_label_4 <- as.factor(df_snf_phenotypes$SNF_label_4) 
df_snf_phenotypes$SNF_label_2 <- as.factor(df_snf_phenotypes$SNF_label_2)
```

### Rewrite male/female as 0/1

```
df_snf_phenotypes$Sex[df_snf_phenotypes$Sex == 'Male'] <- 0
df_snf_phenotypes$Sex[df_snf_phenotypes$Sex == 'Female'] <- 1
df_snf_phenotypes$Sex <- as.numeric(df_snf_phenotypes$Sex)
```

# GEE models with 4 SNF clusters as predictor

### Aggression score, Age and Sex as outcome

```
geemodel_agg <- run_geemodel(df_snf_phenotypes, 'Aggression_Tscore', 'SNF_label_4')
geemodel_age <- run_geemodel(df_snf_phenotypes, 'Age', 'SNF_label_4')
geemodel_sex <- run_geemodel(df_snf_phenotypes, 'Sex', 'SNF_label_4', 'binomial')
```

GEE model with Aggression T-score as outcome


|  | Estimate | Naive.S.E. | Naive.z | Robust.S.E. | Robust.z | pval | padjusted |
| --- | --- | --- | --- | --- | --- | --- | --- |
| (Intercept) | 52.0558379 | 0.5558342 | 93.6535404 | 0.5825707 | 89.3554042 | 0.0000000 | 0.0000000 |
| SNF\_label\_4SNF\_1 | 0.0331735 | 0.7767149 | 0.0427100 | 0.7606449 | 0.0436123 | 0.9652135 | 1.0000000 |
| SNF\_label\_4SNF\_2 | -0.9686044 | 1.0185372 | -0.9509760 | 0.7995511 | -1.2114352 | 0.2257286 | 0.6771859 |
| SNF\_label\_4SNF\_3 | -0.2162909 | 0.8609907 | -0.2512116 | 0.8899187 | -0.2430457 | 0.8079700 | 1.0000000 |

GEE model with Age as outcome


|  | Estimate | Naive.S.E. | Naive.z | Robust.S.E. | Robust.z | pval | padjusted |
| --- | --- | --- | --- | --- | --- | --- | --- |
| (Intercept) | 9.0859181 | 0.0804051 | 113.0017 | 0.0814033 | 111.6160344 | 0.0000000 | 0.0000000 |
| SNF\_label\_4SNF\_1 | 0.0009252 | NaN | NaN | 0.0125712 | 0.0736008 | 0.9413281 | 1.0000000 |
| SNF\_label\_4SNF\_2 | -0.0065958 | NaN | NaN | 0.0162894 | -0.4049125 | 0.6855418 | 1.0000000 |
| SNF\_label\_4SNF\_3 | 0.0603068 | NaN | NaN | 0.0562410 | 1.0722925 | 0.2835887 | 0.8507661 |

GEE model with Sex as outcome


|  | Estimate | Naive.S.E. | Naive.z | Robust.S.E. | Robust.z | pval | padjusted |
| --- | --- | --- | --- | --- | --- | --- | --- |
| (Intercept) | -0.1590829 | 0.0883589 | -1.8004170 | 0.0864617 | -1.8399237 | 0.0657794 | 0.2631178 |
| SNF\_label\_4SNF\_1 | -0.0081028 | 0.0682986 | -0.1186385 | 0.0367946 | -0.2202186 | 0.8257009 | 0.8257009 |
| SNF\_label\_4SNF\_2 | -0.1083750 | 0.0933238 | -1.1612796 | 0.0737571 | -1.4693506 | 0.1417377 | 0.4252131 |
| SNF\_label\_4SNF\_3 | -0.0499806 | 0.0760359 | -0.6573292 | 0.0549224 | -0.9100227 | 0.3628105 | 0.7256211 |

### MCA dimension coordinates as outcome

```
geemodel_mca1 <- run_geemodel(df_snf_phenotypes, 'Dim_1', 'SNF_label_4')
geemodel_mca2 <-run_geemodel(df_snf_phenotypes, 'Dim_2', 'SNF_label_4')
geemodel_mca3 <-run_geemodel(df_snf_phenotypes, 'Dim_3', 'SNF_label_4')
geemodel_mca4 <-run_geemodel(df_snf_phenotypes, 'Dim_4', 'SNF_label_4')
geemodel_mca5 <-run_geemodel(df_snf_phenotypes, 'Dim_5', 'SNF_label_4')
geemodel_mca6 <-run_geemodel(df_snf_phenotypes, 'Dim_6', 'SNF_label_4')
geemodel_mca7 <-run_geemodel(df_snf_phenotypes, 'Dim_7', 'SNF_label_4')
geemodel_mca8 <-run_geemodel(df_snf_phenotypes, 'Dim_8', 'SNF_label_4')
geemodel_mca9 <-run_geemodel(df_snf_phenotypes, 'Dim_9', 'SNF_label_4')
geemodel_mca10 <-run_geemodel(df_snf_phenotypes, 'Dim_10', 'SNF_label_4')
```

### Print GEE models

GEE model with MCA dimension 1 as outcome


|  | Estimate | Naive.S.E. | Naive.z | Robust.S.E. | Robust.z | pval | padjusted |
| --- | --- | --- | --- | --- | --- | --- | --- |
| (Intercept) | 0.0131509 | 0.0262688 | 0.5006283 | 0.0272154 | 0.4832140 | 0.6289438 | 1.0000000 |
| SNF\_label\_4SNF\_1 | -0.0126352 | 0.0383120 | -0.3297965 | 0.0359377 | -0.3515849 | 0.7251496 | 1.0000000 |
| SNF\_label\_4SNF\_2 | -0.0429189 | 0.0499770 | -0.8587731 | 0.0350895 | -1.2231268 | 0.2212818 | 0.8851273 |
| SNF\_label\_4SNF\_3 | -0.0076033 | 0.0424593 | -0.1790737 | 0.0436842 | -0.1740524 | 0.8618243 | 1.0000000 |

GEE model with MCA dimension 2 as outcome


|  | Estimate | Naive.S.E. | Naive.z | Robust.S.E. | Robust.z | pval | padjusted |
| --- | --- | --- | --- | --- | --- | --- | --- |
| (Intercept) | 0.0002496 | 0.0192034 | 0.0129963 | 0.0211277 | 0.0118126 | 0.9905751 | 1 |
| SNF\_label\_4SNF\_1 | -0.0105822 | 0.0366162 | -0.2890032 | 0.0281051 | -0.3765221 | 0.7065288 | 1 |
| SNF\_label\_4SNF\_2 | -0.0250091 | 0.0455109 | -0.5495191 | 0.0327995 | -0.7624859 | 0.4457700 | 1 |
| SNF\_label\_4SNF\_3 | -0.0223538 | 0.0407514 | -0.5485394 | 0.0314699 | -0.7103212 | 0.4775050 | 1 |

GEE model with MCA dimension 3 as outcome


|  | Estimate | Naive.S.E. | Naive.z | Robust.S.E. | Robust.z | pval | padjusted |
| --- | --- | --- | --- | --- | --- | --- | --- |
| (Intercept) | 0.0115625 | 0.0159819 | 0.7234731 | 0.0166449 | 0.6946540 | 0.4872721 | 1 |
| SNF\_label\_4SNF\_1 | -0.0175738 | 0.0277860 | -0.6324684 | 0.0263071 | -0.6680243 | 0.5041181 | 1 |
| SNF\_label\_4SNF\_2 | 0.0025331 | 0.0352258 | 0.0719110 | 0.0255299 | 0.0992220 | 0.9209620 | 1 |
| SNF\_label\_4SNF\_3 | -0.0149622 | 0.0308038 | -0.4857272 | 0.0235227 | -0.6360772 | 0.5247261 | 1 |

GEE model with MCA dimension 4 as outcome


|  | Estimate | Naive.S.E. | Naive.z | Robust.S.E. | Robust.z | pval | padjusted |
| --- | --- | --- | --- | --- | --- | --- | --- |
| (Intercept) | 0.0072812 | 0.0153545 | 0.4742070 | 0.0187810 | 0.3876895 | 0.6982458 | 1 |
| SNF\_label\_4SNF\_1 | -0.0101670 | 0.0292809 | -0.3472245 | 0.0186138 | -0.5462089 | 0.5849223 | 1 |
| SNF\_label\_4SNF\_2 | -0.0020463 | 0.0363927 | -0.0562272 | 0.0258816 | -0.0790623 | 0.9369831 | 1 |
| SNF\_label\_4SNF\_3 | -0.0116309 | 0.0325880 | -0.3569064 | 0.0224595 | -0.5178601 | 0.6045559 | 1 |

GEE model with MCA dimension 5 as outcome


|  | Estimate | Naive.S.E. | Naive.z | Robust.S.E. | Robust.z | pval | padjusted |
| --- | --- | --- | --- | --- | --- | --- | --- |
| (Intercept) | -0.0013544 | 0.0117329 | -0.1154342 | 0.0113085 | -0.1197658 | 0.9046686 | 1 |
| SNF\_label\_4SNF\_1 | -0.0153876 | 0.0187150 | -0.8222087 | 0.0180874 | -0.8507400 | 0.3949138 | 1 |
| SNF\_label\_4SNF\_2 | 0.0086694 | 0.0240975 | 0.3597644 | 0.0168020 | 0.5159746 | 0.6058721 | 1 |
| SNF\_label\_4SNF\_3 | -0.0301414 | 0.0207356 | -1.4536060 | 0.0272162 | -1.1074787 | 0.2680870 | 1 |

GEE model with MCA dimension 6 as outcome


|  | Estimate | Naive.S.E. | Naive.z | Robust.S.E. | Robust.z | pval | padjusted |
| --- | --- | --- | --- | --- | --- | --- | --- |
| (Intercept) | 0.0120335 | 0.0108661 | 1.1074342 | 0.0113323 | 1.0618817 | 0.2882894 | 1 |
| SNF\_label\_4SNF\_1 | -0.0143700 | 0.0196621 | -0.7308495 | 0.0177362 | -0.8102065 | 0.4178215 | 1 |
| SNF\_label\_4SNF\_2 | -0.0013172 | 0.0247218 | -0.0532826 | 0.0210130 | -0.0626869 | 0.9500159 | 1 |
| SNF\_label\_4SNF\_3 | -0.0260293 | 0.0218189 | -1.1929712 | 0.0227576 | -1.1437667 | 0.2527204 | 1 |

GEE model with MCA dimension 7 as outcome


|  | Estimate | Naive.S.E. | Naive.z | Robust.S.E. | Robust.z | pval | padjusted |
| --- | --- | --- | --- | --- | --- | --- | --- |
| (Intercept) | 0.0094431 | 0.0099925 | 0.9450187 | 0.0107856 | 0.8755208 | 0.3812906 | 0.9631224 |
| SNF\_label\_4SNF\_1 | -0.0224932 | 0.0183364 | -1.2266981 | 0.0175946 | -1.2784149 | 0.2011032 | 0.8044127 |
| SNF\_label\_4SNF\_2 | -0.0157695 | 0.0229852 | -0.6860701 | 0.0158915 | -0.9923213 | 0.3210408 | 0.9631224 |
| SNF\_label\_4SNF\_3 | 0.0006456 | 0.0203589 | 0.0317093 | 0.0164186 | 0.0393192 | 0.9686359 | 0.9686359 |

GEE model with MCA dimension 8 as outcome


|  | Estimate | Naive.S.E. | Naive.z | Robust.S.E. | Robust.z | pval | padjusted |
| --- | --- | --- | --- | --- | --- | --- | --- |
| (Intercept) | -0.0049335 | 0.0104314 | -0.4729416 | 0.0110499 | -0.4464725 | 0.6552560 | 1 |
| SNF\_label\_4SNF\_1 | -0.0169534 | 0.0170464 | -0.9945444 | 0.0151494 | -1.1190773 | 0.2631072 | 1 |
| SNF\_label\_4SNF\_2 | 0.0121874 | 0.0218604 | 0.5575129 | 0.0191752 | 0.6355831 | 0.5250482 | 1 |
| SNF\_label\_4SNF\_3 | 0.0242876 | 0.0188875 | 1.2859136 | 0.0257216 | 0.9442510 | 0.3450414 | 1 |

GEE model with MCA dimension 9 as outcome


|  | Estimate | Naive.S.E. | Naive.z | Robust.S.E. | Robust.z | pval | padjusted |
| --- | --- | --- | --- | --- | --- | --- | --- |
| (Intercept) | -0.0132020 | 0.0105128 | -1.2558036 | 0.0110626 | -1.1933900 | 0.2327167 | 0.6981500 |
| SNF\_label\_4SNF\_1 | 0.0304467 | 0.0178862 | 1.7022484 | 0.0155574 | 1.9570620 | 0.0503402 | 0.2013607 |
| SNF\_label\_4SNF\_2 | -0.0060089 | 0.0227707 | -0.2638893 | 0.0148307 | -0.4051702 | 0.6853524 | 1.0000000 |
| SNF\_label\_4SNF\_3 | 0.0052656 | 0.0198231 | 0.2656281 | 0.0234486 | 0.2245580 | 0.8223232 | 1.0000000 |

GEE model with MCA dimension 10 as outcome


|  | Estimate | Naive.S.E. | Naive.z | Robust.S.E. | Robust.z | pval | padjusted |
| --- | --- | --- | --- | --- | --- | --- | --- |
| (Intercept) | -0.0086705 | 0.0100961 | -0.8587897 | 0.0105705 | -0.8202469 | 0.4120754 | 1.0000000 |
| SNF\_label\_4SNF\_1 | 0.0059236 | 0.0183417 | 0.3229576 | 0.0142971 | 0.4143202 | 0.6786396 | 1.0000000 |
| SNF\_label\_4SNF\_2 | 0.0354818 | 0.0230419 | 1.5398808 | 0.0186191 | 1.9056679 | 0.0566933 | 0.2267732 |
| SNF\_label\_4SNF\_3 | 0.0108521 | 0.0203566 | 0.5331021 | 0.0182546 | 0.5944864 | 0.5521868 | 1.0000000 |

# GEE models with 2 SNF clusters as predictor

### Aggression score, Age and Sex as outcome

```
geemodel_agg <- run_geemodel(df_snf_phenotypes, 'Aggression_Tscore', 'SNF_label_2')
geemodel_age <- run_geemodel(df_snf_phenotypes, 'Age', 'SNF_label_2')
geemodel_sex <- run_geemodel(df_snf_phenotypes, 'Sex', 'SNF_label_2', 'binomial')
```

GEE model with Aggression T-score as outcome


|  | Estimate | Naive.S.E. | Naive.z | Robust.S.E. | Robust.z | pval | padjusted |
| --- | --- | --- | --- | --- | --- | --- | --- |
| (Intercept) | 51.9683023 | 0.5164482 | 100.6263533 | 0.5221822 | 99.5214013 | 0.0000000 | 0.0000000 |
| SNF\_label\_2SNF\_1 | -0.2055225 | 0.6983032 | -0.2943169 | 0.7657876 | -0.2683805 | 0.7884064 | 0.7884064 |

GEE model with Age as outcome


|  | Estimate | Naive.S.E. | Naive.z | Robust.S.E. | Robust.z | pval | padjusted |
| --- | --- | --- | --- | --- | --- | --- | --- |
| (Intercept) | 9.0801066 | 0.0811294 | 111.9213 | 0.0813495 | 111.618412 | 0.0000000 | 0.0000000 |
| SNF\_label\_2SNF\_1 | 0.0582866 | NaN | NaN | 0.0405686 | 1.436741 | 0.1507916 | 0.1507916 |

GEE model with Sex as outcome


|  | Estimate | Naive.S.E. | Naive.z | Robust.S.E. | Robust.z | pval | padjusted |
| --- | --- | --- | --- | --- | --- | --- | --- |
| (Intercept) | -0.1805085 | 0.0865074 | -2.0866257 | 0.0860522 | -2.0976620 | 0.0359350 | 0.0718700 |
| SNF\_label\_2SNF\_1 | -0.0000923 | 0.0636263 | -0.0014505 | 0.0383636 | -0.0024057 | 0.9980806 | 0.9980806 |

### MCA dimension coordinates as outcome

```
geemodel_mca1 <- run_geemodel(df_snf_phenotypes, 'Dim_1', 'SNF_label_2')
geemodel_mca2 <-run_geemodel(df_snf_phenotypes, 'Dim_2', 'SNF_label_2')
geemodel_mca3 <-run_geemodel(df_snf_phenotypes, 'Dim_3', 'SNF_label_2')
geemodel_mca4 <-run_geemodel(df_snf_phenotypes, 'Dim_4', 'SNF_label_2')
geemodel_mca5 <-run_geemodel(df_snf_phenotypes, 'Dim_5', 'SNF_label_2')
geemodel_mca6 <-run_geemodel(df_snf_phenotypes, 'Dim_6', 'SNF_label_2')
geemodel_mca7 <-run_geemodel(df_snf_phenotypes, 'Dim_7', 'SNF_label_2')
geemodel_mca8 <-run_geemodel(df_snf_phenotypes, 'Dim_8', 'SNF_label_2')
geemodel_mca9 <-run_geemodel(df_snf_phenotypes, 'Dim_9', 'SNF_label_2')
geemodel_mca10 <-run_geemodel(df_snf_phenotypes, 'Dim_10', 'SNF_label_2')
```

### Print GEE models

GEE model with MCA dimension 1 as outcome


|  | Estimate | Naive.S.E. | Naive.z | Robust.S.E. | Robust.z | pval | padjusted |
| --- | --- | --- | --- | --- | --- | --- | --- |
| (Intercept) | 0.0015172 | 0.0242463 | 0.0625735 | 0.0239219 | 0.0634222 | 0.9494303 | 1 |
| SNF\_label\_2SNF\_1 | 0.0134663 | 0.0343284 | 0.3922798 | 0.0441630 | 0.3049232 | 0.7604247 | 1 |

GEE model with MCA dimension 2 as outcome


|  | Estimate | Naive.S.E. | Naive.z | Robust.S.E. | Robust.z | pval | padjusted |
| --- | --- | --- | --- | --- | --- | --- | --- |
| (Intercept) | -0.0205546 | 0.0164944 | -1.246162 | 0.0126323 | -1.627150 | 0.1037052 | 0.2074104 |
| SNF\_label\_2SNF\_1 | 0.0530564 | 0.0323396 | 1.640601 | 0.0505972 | 1.048605 | 0.2943601 | 0.2943601 |

GEE model with MCA dimension 3 as outcome


|  | Estimate | Naive.S.E. | Naive.z | Robust.S.E. | Robust.z | pval | padjusted |
| --- | --- | --- | --- | --- | --- | --- | --- |
| (Intercept) | 0.0026467 | 0.0141759 | 0.1867019 | 0.0133481 | 0.1982798 | 0.8428261 | 1 |
| SNF\_label\_2SNF\_1 | 0.0155254 | 0.0246786 | 0.6291041 | 0.0307035 | 0.5056562 | 0.6130980 | 1 |

GEE model with MCA dimension 4 as outcome


|  | Estimate | Naive.S.E. | Naive.z | Robust.S.E. | Robust.z | pval | padjusted |
| --- | --- | --- | --- | --- | --- | --- | --- |
| (Intercept) | 0.0079058 | 0.0132105 | 0.598449 | 0.0141267 | 0.5596334 | 0.5757295 | 0.63375 |
| SNF\_label\_2SNF\_1 | -0.0187002 | 0.0258876 | -0.722362 | 0.0186834 | -1.0009003 | 0.3168750 | 0.63375 |

GEE model with MCA dimension 5 as outcome


|  | Estimate | Naive.S.E. | Naive.z | Robust.S.E. | Robust.z | pval | padjusted |
| --- | --- | --- | --- | --- | --- | --- | --- |
| (Intercept) | -0.0066474 | 0.0106456 | -0.6244249 | 0.0100420 | -0.6619541 | 0.5080007 | 1 |
| SNF\_label\_2SNF\_1 | -0.0040758 | 0.0167314 | -0.2436048 | 0.0206443 | -0.1974314 | 0.8434899 | 1 |

GEE model with MCA dimension 6 as outcome


|  | Estimate | Naive.S.E. | Naive.z | Robust.S.E. | Robust.z | pval | padjusted |
| --- | --- | --- | --- | --- | --- | --- | --- |
| (Intercept) | 0.0081686 | 0.0095317 | 0.8569937 | 0.0098890 | 0.8260301 | 0.4087870 | 0.817574 |
| SNF\_label\_2SNF\_1 | -0.0115848 | 0.0174355 | -0.6644405 | 0.0170602 | -0.6790545 | 0.4971034 | 0.817574 |

GEE model with MCA dimension 7 as outcome


|  | Estimate | Naive.S.E. | Naive.z | Robust.S.E. | Robust.z | pval | padjusted |
| --- | --- | --- | --- | --- | --- | --- | --- |
| (Intercept) | 0.0007598 | 0.0087380 | 0.0869524 | 0.0081773 | 0.0929143 | 0.9259717 | 1 |
| SNF\_label\_2SNF\_1 | 0.0112585 | 0.0162463 | 0.6929860 | 0.0211619 | 0.5320155 | 0.5947153 | 1 |

GEE model with MCA dimension 8 as outcome


|  | Estimate | Naive.S.E. | Naive.z | Robust.S.E. | Robust.z | pval | padjusted |
| --- | --- | --- | --- | --- | --- | --- | --- |
| (Intercept) | -0.0037300 | 0.0094025 | -0.3967056 | 0.0092691 | -0.4024128 | 0.6873803 | 1 |
| SNF\_label\_2SNF\_1 | 0.0022459 | 0.0152267 | 0.1474997 | 0.0188424 | 0.1191959 | 0.9051202 | 1 |

GEE model with MCA dimension 9 as outcome


|  | Estimate | Naive.S.E. | Naive.z | Robust.S.E. | Robust.z | pval | padjusted |
| --- | --- | --- | --- | --- | --- | --- | --- |
| (Intercept) | -0.0083145 | 0.0093877 | -0.8856735 | 0.0092541 | -0.8984662 | 0.3689371 | 0.7378741 |
| SNF\_label\_2SNF\_1 | 0.0037702 | 0.0159404 | 0.2365199 | 0.0179414 | 0.2101410 | 0.8335576 | 0.8335576 |

GEE model with MCA dimension 10 as outcome


|  | Estimate | Naive.S.E. | Naive.z | Robust.S.E. | Robust.z | pval | padjusted |
| --- | --- | --- | --- | --- | --- | --- | --- |
| (Intercept) | 0.0029445 | 0.0088353 | 0.3332639 | 0.0089680 | 0.3283343 | 0.7426589 | 0.7426589 |
| SNF\_label\_2SNF\_1 | -0.0203261 | 0.0162612 | -1.2499711 | 0.0145899 | -1.3931586 | 0.1635718 | 0.3271436 |

```
sessionInfo()
```

```
## R version 4.0.2 (2020-06-22)
## Platform: x86_64-pc-linux-gnu (64-bit)
## Running under: Debian GNU/Linux bullseye/sid
## 
## Matrix products: default
## BLAS:   /usr/lib/x86_64-linux-gnu/openblas-pthread/libblas.so.3
## LAPACK: /usr/lib/x86_64-linux-gnu/openblas-pthread/libopenblasp-r0.3.10.so
## 
## locale:
##  [1] LC_CTYPE=en_US.UTF-8       LC_NUMERIC=C              
##  [3] LC_TIME=en_US.UTF-8        LC_COLLATE=en_US.UTF-8    
##  [5] LC_MONETARY=en_US.UTF-8    LC_MESSAGES=en_US.UTF-8   
##  [7] LC_PAPER=en_US.UTF-8       LC_NAME=C                 
##  [9] LC_ADDRESS=C               LC_TELEPHONE=C            
## [11] LC_MEASUREMENT=en_US.UTF-8 LC_IDENTIFICATION=C       
## 
## attached base packages:
## [1] stats     graphics  grDevices utils     datasets  methods   base     
## 
## other attached packages:
## [1] gee_4.13-25
## 
## loaded via a namespace (and not attached):
##  [1] digest_0.6.31   R6_2.5.1        jsonlite_1.8.4  evaluate_0.20  
##  [5] rlang_1.1.0     cachem_1.0.7    cli_3.6.1       jquerylib_0.1.4
##  [9] bslib_0.4.2     rmarkdown_2.21  tools_4.0.2     xfun_0.38      
## [13] yaml_2.3.7      fastmap_1.1.1   compiler_4.0.2  htmltools_0.5.5
## [17] knitr_1.42      sass_0.4.5
```

---

1. Radboud University Medical Center, casper.devisser@radboudumc.nl↩︎
