# Supplementary material for: A multi-omics data analysis workflow packaged as a FAIR Digital Object
Source: Gigascience. 2024 Jan 13;13:giad115. doi: 10.1093/gigascience/giad115 (PMC10787363; doi:10.1093/gigascience/giad115)
Supplement: giad115_Response_to_Reviewer_Comments_Original_Submission [file giad115_response_to_reviewer_comments_original_submission.pdf]

Author's Response To Reviewer Comments

Please find our rebuttal uploaded as supplementary file
