# Supplementary material for: A multi-omics data analysis workflow packaged as a FAIR Digital Object
Source: Gigascience. 2024 Jan 13;13:giad115. doi: 10.1093/gigascience/giad115 (PMC10787363; doi:10.1093/gigascience/giad115)
Supplement: giad115_Reviewer_1_Report_Original_Submission — Carole Goble -- 7/13/2023 Reviewed [file giad115_reviewer_1_report_original_submission.pdf]

Reviewer Report

Title: A Multi-omics Data Analysis Workflow Packaged as a FAIR Digital Object

Version: Original Submission **Date:** 7/13/2023

Reviewer name: Carole Goble

Reviewer Comments to Author:

This work reports a multi-omics data analysis workflow packaged as a RO-Crate, an implementation of a FAIR Digital Object.We limit our comments to the technical aspects of the Research Object and workflow packaging. The scientific validity of the omics analysis itself is outside our expertise.The paper is comprehensive and the background grounding in the current state of the art is excellent and thorough. The paper is an excellent exemplar of the future of data analysis reporting for FAIR and reproducible computational methods, and the amount of work impressive. We congratulate the authors.WorkflowHub entry https://workflowhub.eu/workflows/402?version=5# gives a comprehensive report of the Nextflow workflow and its multiple versions, all the files including the R scripts and the synthetic data. The RO-Crate rendering looks correct and version-locking the R containers is following best practice(https://github.com/Xomics/ACTIONdemonstrator_workflow/blob/main/nextflow.config#L44)The paper also highlights the amount of work needed to make such a pipeline to be both metadata machine processable and metadata human readable.To make this pipeline reproducible requires a mixture of notebooks submitted as supplementary materials, the Nextflow workflow with its R scripts represented as an RO-Crate in WorkflowHub and a README is linked to the container recipes in https://github.com/Xomics/Docker_containers and then another Documentation.md file. There seems to be the potential for duplicated effort in reporting the necessary metadata describing the workflow that could be highlighted in the Discussion as a steer to the digital object community.- Could the RO-Crate approach be widened beyond the current Workflow RO-Crate, and would there be value in streamlining the metadata, or is this just an artefact of the need for multiple descriptions and ease of publishing. If the JSON within the RO-Crate was more richly annotated, could some of the Documentation.md be avoided altogether, and is that even desirable?- The README includes the container/software packaging and is not linked from the RO-Crate (and there isn't an obvious property to link to it yet). Could these be RO-Crates too?- The notebooks in the supplementary files could also be registered in WorkflowHub and linked to the Nextflow workflow (see https://workflowhub.eu/workflows?filter%5Bworkflow_type%5D=jupyter).- Is it feasible and desirable to have a single RO-Crate linked to many other RO-Crates to represent the whole reproducible pipeline in full?In the discussion the FAIR principles verification through different practices and approaches would be more helpful if it was more precise. Comments seem to be limited to the Workflow RO-Crate and use of ontologies for machine readability. As highlighted in table 1 there is more to FAIR software & workflows than this.Minor remarksKey points- We here demonstrate the implementation multi-omics data -> We here demonstrate an implementation of an multi-omics data.Background- The documentation of dependencies is highlighted as a prerequisite for software interoperability. In the

FAIR4RS principles I2 also highlights qualified references to other objects - presumably other software or installation requirements. This highlights the relationship between software interoperability and software portability. It seems that dependencies more relate to portability rather than interoperability.- "Based on the FDO concept, the RO-Crate approach was specified". This is a confusing statement. RO-Crates have been recognised as an implementation approach for the FDO concept as proposed by the FDO Forum. For more discussion on FDO and the Linked Data approach promoted by RO-Crates see https://arxiv.org/abs/2306.07436. However, RO-Crates are not based in the FDO - they are based on the Research Object packaging work that emerged from the EU Wf4ever project, (see https://doi.org/10.1016/j.future.2011.08.004 from 2013).- It is better to describe the RO-Crate metadata file as " It contains all contextual and non-contextual related data to re-run the workflow". Instead of "It can additionally contain data on which the workflow can be run."Workflow Implementation- At the beginning of the last paragraph, "Besides the workflow and the synthetic data set" replace with "As well as the workflow and the synthetic data set".- https://workflowhub.eu/workflows/402?version=5# gives a very nice pictorial overview of the workflow that you may consider including in the paper itself.

Level of Interest

Please indicate how interesting you found the manuscript: Choose an item.

Quality of Written English

Please indicate the quality of language in the manuscript: Choose an item.

Declaration of Competing Interests

Please complete a declaration of competing interests, considering the following questions:

- Have you in the past five years received reimbursements, fees, funding, or salary from an organisation that may in any way gain or lose financially from the publication of this manuscript, either now or in the future?
- Do you hold any stocks or shares in an organisation that may in any way gain or lose financially from the publication of this manuscript, either now or in the future?
- Do you hold or are you currently applying for any patents relating to the content of the manuscript?
- Have you received reimbursements, fees, funding, or salary from an organization that holds or has applied for patents relating to the content of the manuscript?
- Do you have any other financial competing interests?
- Do you have any non-financial competing interests in relation to this paper?

If you can answer no to all of the above, write 'I declare that I have no competing interests' below. If your reply is yes to any, please give details below.

I declare that I have no competing interests

I agree to the open peer review policy of the journal. I understand that my name will be included on my report to the authors and, if the manuscript is accepted for publication, my named report including any attachments I upload will be posted on the website along with the authors' responses. I agree for my report to be made available under an Open Access Creative Commons CC-BY license (http://creativecommons.org/licenses/by/4.0/). I understand that any comments which I do not wish to be included in my named report can be included as confidential comments to the editors, which will not be published.

Choose an item.

To further support our reviewers, we have joined with Publons, where you can gain additional credit to further highlight your hard work (see: https://publons.com/journal/530/gigascience). On publication of this paper, your review will be automatically added to Publons, you can then choose whether or not to claim your Publons credit. I understand this statement.

Yes Choose an item.
