# Supplementary material for: A multi-omics data analysis workflow packaged as a FAIR Digital Object
Source: Gigascience. 2024 Jan 13;13:giad115. doi: 10.1093/gigascience/giad115 (PMC10787363; doi:10.1093/gigascience/giad115)
Supplement: giad115_Reviewer_3_Report_Original_Submission — Megan Hagenauer, Ph.D. -- 8/31/2023 Reviewed [file giad115_reviewer_3_report_original_submission.pdf]

Reviewer Report

Title: A Multi-omics Data Analysis Workflow Packaged as a FAIR Digital Object

Version: Original Submission **Date:** 8/31/2023

Reviewer name: Megan Hagenauer, Ph.D.

Reviewer Comments to Author:

Review of "A Multi-omics Data Analysis Workflow Packaged as a FAIR Digital Object" by Niehues et al. for GigaScience08-31-2023I want to begin by apologizing for the tardiness of this review - my whole family caught Covid during the review period, and it has taken several weeks for us to be functional again.OverviewAs a genomics data analyst, I found this manuscript to be a fascinating, inspiring, and, quite honestly, intimidating, view into the process of making analysis code and workflow truly meet FAIR standards. I have added recommendations below for elements to add to the manuscript that would help myself and other analysts use your case study to plan out our own workflows and code release. These recommendations fall quite solidly into the "Minor Revision" category and may require some editorial oversight as this article type is new to me. Please note that I only had access to the main text of the manuscript while writing this review.Specific Comments1) As a case study, it would be useful to have more explicit discussion of the expertise and effort involved in the FAIR code release and the anticipated cost/benefit ratio:As a data analyst, I have a deep, vested interest in reproducible science and improved workflow/code reusability, but also a limited bandwidth. For me, your overview of the process of producing a FAIR code release was both inspiring and daunting, and left me with many questions about the feasibility of following in your footsteps. The value of your case study would be greatly enhanced by discussing cost/benefit in more detail:a. What sort of expertise or training was required to complete each step in the FAIR release? E.g.,i. Was your use of tools like Github, Jupyter notebook, WorkflowHub, and DockerHub something that could be completed by a scientist with introductory training in these tools, or did it require higher level use?ii. Was there any particular training required for the production of high quality user documentation or metadata? (e.g., navigating ontologies?)b. With this expertise/training in place, how much time and effort do you estimate that it took to complete each step of adapting your analysis workflow and code release to meet FAIR standards?i. Do you think this time and effort would differ if an analyst planned to meet FAIR standards for analysis code prior to initiating the analysis versus deciding post-hoc to make the release of previously created code fit FAIR standards?c. The introduction provides an excellent overview of the potential benefits of releasing FAIR analysis code/workflows. How did these benefits end up playing out within your specific case study?i. e.g., I thought this sentence in your discussion was a particularly important note about the benefits of FAIR analysis code in your study: "Developing workflows with partners across multiple institutions can pose a challenge and we experienced that a secure shared computing environment was key to the success of this project."ii. Has the FAIR analysis workflow also been useful for collaboration or training in your lab?iii. How many of the analysis modules (or other aspects of the pipeline) do you plan on reusing? In general, what do you think is the size for the audience for reuse of the FAIR code? (e.g., how many people do you think will have been saved significant amounts of work by you putting in this

effort?)iv. … Or is the primary benefit mostly just improving the transparency/reproducibility of your science?d. If there is any way to easily overview these aspects of your case study (effort/time, expertise, immediate benefits) in a table or figure, that would be ideal. This is definitely the content that I would be skimming your paper to find.2) As a reusable code workflow, it would be useful to provide additional information about the data input and experimental design, so that readers can determine how easily the workflow could be adapted to their own datasets. This information could be added to the text or to Fig 1. E.g.,i. The dimensionality of the input (sample size, number of independent variables & potential co-variates, number of dependent variables in each dataset, etc)ii. Data types for the independent variables, co-variates, and dependent variables (e.g., categorical, numeric, etc)iii. Any collinearity between independent variables (e.g., nesting, confounding).3) As documentation of the analysis, it would be useful to provide additional information about how the analysis workflow may influence the interpretation of the results.a. It would be especially useful to know which aspects of the analysis were preplanned or following a standard procedure/protocol, and which aspects of the analysis were customized after reviewing the data or results. This information can help the reader assess the risk of overfitting or HARKing.b. It would also be useful to call out explicitly how certain analysis decisions change the interpretation of the results. In particular, the decision to use dimension reduction techniques within the analysis of both the independent and dependent variables, and then focus only on the top dimensions explaining the largest sources of variation within the datasets, is especially important to justify and describe its impact on the interpretation of the results. Is there reason to believe that externalizing behavior should be related to the largest sources of variation within buccal DNA methylation or urinary metabolites? Within genetic analyses, the assumption tends to be the opposite - that genetic variation related to behavior (such as externalizing) is likely to be present in a small percent of the genome, and that the top sources of variation within the genetics dataset are uninteresting (related to population) and therefore traditionally filtered out of the data prior to analysis. Within transcriptomics, if a tissue is involved in generating the behavior, some of the top dimensions explaining the largest sources of variation in the dataset may be related to that behavior, but the absolute largest sources of variation are almost always technical artifacts (e.g., processing batches, dissection batches) or impactful sources of biological noise (e.g., age, sex, cell type heterogeneity in the tissue). Is there reason to believe that cheek cells would have their main sources of epigenetic variation strongly related to externalizing behavior? (maybe as a canary in a coal mine for other whole organism events like developmental stress exposure?). Is there reason to believe that the primary variation in urinary metabolites would be related to externalizing behavior? (perhaps as a stand-in for other large-scale organismal states that might be related to the behavior - hormonal states? metabolic states? inflammation?). Since the goal of this paper is to provide a case study for creating a FAIR data analysis workflow, it is less important that you have strong answers for these questions, and more important that you are transparent about how the answers to these questions change the interpretation of your results. Adding a few sentences to the discussion is probably sufficient to serve this purpose.Thank you for your hard work helping advance our field towards greater transparency and reproducibility. I look forward to seeing your paper published so that I can share it with the other analysts in our lab.

Level of Interest

Please indicate how interesting you found the manuscript: Choose an item.

Quality of Written English

Please indicate the quality of language in the manuscript: Choose an item.

Declaration of Competing Interests

Please complete a declaration of competing interests, considering the following questions:

- Have you in the past five years received reimbursements, fees, funding, or salary from an organisation that may in any way gain or lose financially from the publication of this manuscript, either now or in the future?
- Do you hold any stocks or shares in an organisation that may in any way gain or lose financially from the publication of this manuscript, either now or in the future?
- Do you hold or are you currently applying for any patents relating to the content of the manuscript?
- Have you received reimbursements, fees, funding, or salary from an organization that holds or has applied for patents relating to the content of the manuscript?
- Do you have any other financial competing interests?
- Do you have any non-financial competing interests in relation to this paper?

If you can answer no to all of the above, write 'I declare that I have no competing interests' below. If your reply is yes to any, please give details below.

I declare that I have no competing interests.

I agree to the open peer review policy of the journal. I understand that my name will be included on my report to the authors and, if the manuscript is accepted for publication, my named report including any attachments I upload will be posted on the website along with the authors' responses. I agree for my report to be made available under an Open Access Creative Commons CC-BY license (http://creativecommons.org/licenses/by/4.0/). I understand that any comments which I do not wish to be included in my named report can be included as confidential comments to the editors, which will not be published.

Choose an item.

To further support our reviewers, we have joined with Publons, where you can gain additional credit to further highlight your hard work (see: https://publons.com/journal/530/gigascience). On publication of this paper, your review will be automatically added to Publons, you can then choose whether or not to claim your Publons credit. I understand this statement.

Yes Choose an item.
